# Molecular Screening for Digital Dermatitis-Associated Treponemes in Bovine Ischaemic Teat Necrosis Lesions and Milk in Dairy Cattle

**DOI:** 10.3390/pathogens13050427

**Published:** 2024-05-17

**Authors:** Hayley E. Crosby-Durrani, Stuart D. Carter, Roger W. Blowey, Nicholas J. Evans

**Affiliations:** 1 Institute of Infection, Veterinary, and Ecological Sciences, University of Liverpool, Leahurst Campus, Chester High Road, Neston CH64 7TE, UK; 2 Appithorne, Main Road, Minsterworth GL2 8JG, UK

**Keywords:** treponemes, dairy cattle, digital dermatitis, ischaemic teat necrosis, milk

## Abstract

Bovine ischaemic teat necrosis (ITN) is a disease affecting the skin of the teats of dairy cows with an unknown aetiopathogenesis. Digital dermatitis (DD)-associated treponemes have previously been suggested as a potential aetiological agent in ITN, although the sample size was small. The current study, using established PCR techniques, aimed to examine the association with the presence of DD-associated treponemes in a large number of ITN samples from a wider geographical area, and surveyed the potential of milk as an infection reservoir. From 95 ITN lesions, 35.8% (*n* = 34) were positive for at least one DD-associated treponeme compared with only 5.6% (*n* = 1) of 18 non-lesioned teats from cows with ITN lesions on a different teat using a nested PCR approach. All 10 age- and production-matched control cows were negative for DD-associated treponemes via PCR. No DD-associated treponemes could be detected from foremilk of cows with (*n* = 19) and without (*n* = 31) a DD lesion on the hind feet. DD-associated treponemes could be detected via PCR after incubation in milk for up to 2 h. Therefore, milk does not appear to be a competent reservoir for transmission of DD-associated treponemes. Moreover, in the current study DD-associated treponemes were only detected in a subset of ITN samples, so it is unlikely these opportunistic skin-associated pathogens are the major or sole agent of ITN.

## 1. Introduction

Bovine ischaemic teat necrosis (ITN) occurs in dairy cattle and is a disease that affects the skin of the teat, notably around the teat base [1,2]. The disease usually occurs in first lactation in dairy cows within the first 30 days in milk and up to the first 90 days in milk [3]. A pilot study previously identified an association with digital dermatitis (DD)-associated treponemes which were detected by PCR in 11 of 12 ITN lesions [1]. This initial study suggested a causative link for DD-associated treponemes and it is timely to clarify this association by surveying a greater number of dairy cattle with ITN from a larger geographical area in the United Kingdom (UK). Digital dermatitis is an infectious foot disease in cattle with an estimated minimum cost of €391.80 per affected cow per year for the dairy industry [4]. There is increasing evidence for the spirochaetal bacteria, pathogenic DD-associated treponemes: *Treponema medium* (*T. medium*), *Treponema phagedenis* (*T. phagedenis*) and *Treponema pedis* (*T. pedis*), as being involved in the aetiopathogenesis of DD [5,6,7,8]. Other bacteria such as *Fusobacterium* sp., *Bacteroides* sp. and *Porphyromonas* sp. are also frequently associated, suggesting that DD is a polymicrobial disease [9,10,11,12]. The DD-associated treponemes have also been detected in a wide range of skin diseases in livestock and wildlife including contagious ovine digital dermatitis (CODD) in sheep [13], a similar presentation in goats [14], wild North American elk [15,16], and skin disease in pigs [17,18,19]. DD-associated treponeme phylogroups have also been detected in another skin lesion of the udder, udder cleft dermatitis (UCD), although only in approximately 10% of cases [20,21]; in the foremilk from cows with UCD and/or DD lesions [22] and in skin lesions of the hocks of dairy cattle [23]. 

When an animal with a DD lesion lies down, the feet can come into contact with the udder, which may allow microbes to be transferred from feet to udder. In addition, while lying, parts of the udder and teats may become compressed, resulting in a moist and anaerobic environment suitable for growth of DD-associated treponemes. Moreover, it is considered that DD-associated treponemes may migrate along the skin to areas of poor skin integrity [23], such as an area of necrotic skin. 

DD-associated treponemes have also been detected in various aspects of the dairy environment, including: in slurry and cow faeces [24]; on hoof knifes [25] and foot trimming equipment [26]; in cow hoof footprints from the floor, and in gloves worn by foot trimmers [27]. Bell et al. [27] also found that DD-associated treponemes could survive in different types of bedding used in the dairy farm by using in vitro experiments that added cultured DD-associated treponemes to clean bedding samples. As these bacteria have also been detected in the foremilk of cows with UCD lesions [22], we investigated in vitro detection of DD-associated treponemes in milk incubated at body temperature (37 °C) and bulk milk tank temperature (4 °C). It was considered relevant to determine if cows presenting with DD lesions on the hind feet had DD-associated treponemes present in the foremilk, as reported in UCD-positive animals. If this was the case, then there would be further implications around the control of DD and another infection reservoir to consider.

The aims of this study were to investigate the presence of DD-associated treponemes in a larger number of ITN samples from a wide geographical area and to broaden the understanding of potential reservoirs for DD-associated treponemes, especially within milk.

## 2. Materials and Methods

### 2.1. Ethics Statement

Ethical approval for this study was granted by the University of Liverpool, School of Veterinary Science Ethical Committee (application number: VREC 460). The participants provided their written informed consent to participate in this study and allowed for samples to be retained and used for research purposes.

### 2.2. Sample Collection

#### 2.2.1. Tissue Samples

Udder tissue samples were provided by veterinary surgeons (VS), throughout Great Britain, debriding or surgically removing lesions from live animals for treatment and control purposes (*n* = 24). Debrided tissue was placed into a sterile container and stored frozen awaiting delivery or collection. Other teat tissue samples (*n* = 9 with ITN) were obtained from animals that presented as cull cows at meat inspection in abattoirs and in fallen stock centres. Cull cow teats were removed from the carcass using sterile scalpel blades; 20 normal teats from healthy animals were taken as negative controls. The teats were halved longitudinally through the lesion. One half was stored on ice prior to freezing at −20 °C for microbiological studies and the other half placed in 10% neutral buffered formalin to be used in another study [2].

#### 2.2.2. Swab Samples

Where tissue samples were not obtainable, the VS obtained plain swabs from the relevant necrotic areas of the teat lesions. For swabbing healthy teats, the swab was first wetted with sterile saline. There were 62 swabs of ITN lesions from 32 animals obtained along with 18 swabs from non-affected teats of the same affected animals. When possible, affected animals were matched with unaffected cows from the same farm at a similar age and stage of production and their healthy teats were swabbed at the same site where ITN lesions develop (*n* = 10 cows). Swabs were stored frozen at −20 °C and transported on ice to the laboratory.

### 2.3. Collection of Milk for Assessment of Treponeme Detection in Milk

Whole milk and whole ultra-high temperature (UHT) milk were obtained from the supermarket for investigations into the ability for DD-associated treponemes to be detected after incubation in milk.

For investigating the presence of treponemes in foremilk, foremilk was taken immediately prior to milking the cow and a simplified DD status (acute, chronic, no DD) of the cow was recorded by use of an inspection mirror in the parlour. The university dairy farm was used for this sample collection due to its close proximity to the laboratory. Samples were immediately processed for DNA extractions and inoculated into oral treponeme enrichment broth (OTEB, Anaerobe systems, Morgan Hill, CA, USA) supplemented with 10% (*v*/*v*) foetal calf serum (FCS) within 2 h after milking.

### 2.4. Detection of Digital Dermatitis Treponemes in Milk

DD-associated treponeme bacteria were used to investigate treponeme detection in milk. For ease of use the *T. phagedenis* phylogroup strain T320A and *T. pedis* phylogroup strain T3552B, stains previously isolated by this laboratory, were utilised. T320A and T3552B were previously stored in 10% (*v*/*v*) glycerol at −80 °C and were thawed and transferred to an anaerobic cabinet under the following conditions: 85% N_2_, 10% H_2_ and 5% CO_2_ at 37 °C. Once thawed, approximately 300 µL (10 drops from a sterile glass Pasteur pipette) was inoculated into OTEB (Anaerobe Systems, Morgan Hill, CA, USA) with 10% (*v*/*v*) FCS [25]. Inocula growth was assessed by spirochete presence and motility under phase contrast microscopy on days 4 and 7 after inoculation. If growth was adequate, the cultures were subsequently subcultured taking approximately 90 µL (3 drops) and passaged into new OTEB 10% (*v*/*v*) FCS every 7 days.

### 2.5. DNA Extraction

#### 2.5.1. Extraction from Swabs and Tissue of ITN Lesions

Approximately 0.5 g of swabs and tissue samples obtained from ITN lesions were finely chopped using a sterile scalpel blade and placed into an Eppendorf tube. DNA was extracted using an established commercial extraction kit, DNeasy Blood and Tissue Kit (Qiagen, Manchester, UK) as per manufacturer instructions [6,14,15,23,27].

#### 2.5.2. Extraction from Liquid Culture

Samples inoculated into OTEB liquid culture and treponeme culture stocks serving as positive controls underwent extraction using the Chelex extraction method [25]. Briefly, a 5% Chelex resin solution was formulated by dissolving 0.5 g Bio-Rad BT Chelex^®^ 100 Resin (Bio-Rad, Watford, UK) in 10 mL distilled water. Approximately 1.5 mL of liquid culture was removed from the OTEB tubes and placed into a clip-lock Eppendorf tube and the sample centrifuged at 13,000 RPM for 5 min and approximately 800 mL of the supernatant removed. The pellet was then re-suspended in the remaining supernatant and 250 µL of the 5% Chelex resin added. The sample and Chelex solution were suspended in a boiling water bath for 10 min before centrifuging at 13,000 RPM for 10 min. The supernatant was placed in a new Eppendorf tube and frozen at −20 °C until required.

#### 2.5.3. Extraction from Milk

Extraction of DNA from milk was performed using the Milk Bacterial DNA Isolation kit (Norgen Biotech Corp., Thorold, ON, Canada) according to manufacturer’s instructions, which utilises enzymatic and chemical lysis of bacteria.

#### 2.5.4. Assessing Quality of Extracted DNA

After extraction, the quality and quantity of DNA was assessed using a NanoDrop^TM^ 2000 Spectrophotometer (Thermo Scientific^TM^, Waltham, MA, USA).

### 2.6. Polymerase Chain Reaction

#### 2.6.1. Nested PCR for Detection of Digital Dermatitis Treponemes

A nested PCR approach was used for the detection of DD-associated treponemes and was performed on all samples in triplicate with DNA extracted from an appropriate DD-associated treponeme phylogroup liquid culture as a positive control, and nuclease free water and the appropriate non-targeted DD-associated phylogroups as negative controls for each assay. The assays were a two-step process with the initial PCR step a universal bacterial 16S rRNA gene amplification [28] and then a second DD-associated treponeme phylogroup specific amplification step [6]. The PCR primers used in each step are listed in Table 1. Both steps utilised the PCR reaction mix: 13.8 µL water, 0.6 µL forward primer (100 pmol/µL), 0.6 µL reverse primer (100 pmol/µL), 4 µL 5 × FIREpol^®^ Ready to Load Master Mix (7.5 mM MgCl_2_) (Solis BioDyne, Tartu, Estonia), 1 µL template DNA. The template DNA for the second step was the product from the first universal bacterial 16S rRNA gene amplification. For each PCR assay, different PCR cycling conditions were required. PCR cycling conditions were specified for 16S rRNA gene, DD-associated *T. medium* phylogroup, *T. phagedenis* phylogroup and *T. pedis* phylogroup (Table 2).

#### 2.6.2. PCR for Detection of Treponema Genus Specific 16S rRNA Gene

In addition to the nested PCR approach, all samples were also screened targeting the *Treponema* genus specific 16S rRNA gene (Table 1) [29]. The PCR reaction mix for the *Treponeme* genus assay was: 13.8 µL water, 0.6 µL forward primer (100 pmol/µL), 0.6 µL reverse primer (100 pmol/µL), 4 µL 5 × FIREpol^®^ Ready to Load Master Mix (12.5 mM MgCl_2_) (Solis BioDyne, Tartu, Estonia), 1 µL template DNA. The PCR reaction cycling conditions differed from the nested approach and are presented in Table 2.

### 2.7. Validation of PCR

The samples obtained in the pilot study [1] were re-processed using the above DNA extraction method and DD-associated treponeme PCR assays to validate this study.

### 2.8. Assessment of Treponeme Detection in Milk

Two processes were used to investigate the ability of DD treponemes to survive in milk. The first was to see if treponemes could be detected by PCR after incubation over different time points. The second was to use phase contrast to look for treponemes at various timepoints after an initial incubation of treponemes in milk at 4 °C and 37 °C for 24 h and then sub-culture at 24 h (see below for details).

Whole milk and whole UHT milk were obtained from the supermarket for investigations into the ability for DD treponemes to survive in milk. DD-associated *T. phagedenis* phylogroup strain T320A and DD-associated *T. pedis* phylogroup strain T3552B were cultured and 6 drops of a known concentration corresponding to 1.14 × 10^8^ treponemal organisms/mL (0.43 optical density at 540 nm wavelength) were spiked into 1 mL of milk and incubated under different conditions.

Initially serial 1 in 10 dilutions of the bacteria were used to assess the impact of different DNA extraction kits on the PCR detection of low numbers of treponemes present in the sample.

Treponemes were added to the milk types to see for how long DNA persisted and could be successfully extracted and detected by PCR methods after incubation in an anaerobic environment at 37 °C. Samples were taken for DNA extraction using Milk Bacterial DNA Isolation kit (Norgen Biotech Corp., Thorold, ON, Canada) according to manufacturer instructions, at the following time points: 10 min, 30 min, 1 h, 2 h, 4 h, 8 h, and 24 h post treponeme inoculation.

After the incubation, the effect of temperature on treponeme DNA integrity was also assessed. Treponemes were spiked into milk and incubated either at 4 °C (to represent storage in a refrigerated bulk milk tank) or at 37 °C (to represent body temperature) for 24 h, DNA was extracted from the milk. For positive controls, treponemes were spiked into OTEB (instead of milk) and then followed the same protocol. The negative control consisted of OTEB being spiked into milk. Additionally, 0.5 mL of the milk/treponeme emulsion was inoculated into OTEB 10% (*v*/*v*) FCS. A sample (1 drop) of the liquid culture was taken at 24 h, 1 week, 2 week, 3 weeks, 4 weeks, 5 weeks and 6 weeks [25] and assessed under phase contrast microscopy (Leitz diaplam microscope with a 40× phase contrast objective, 400× magnification) to identify treponeme morphology and motility. After 6 weeks incubation in an anaerobic cabinet a Chelex extraction was performed on a sample of the liquid culture and the extracted DNA analysed by the DD-associated treponeme PCR assays. This process was repeated with two strains of treponemes (T320A and T3552B) with three independent experimental replicates and three technical replicates.

The above process was also repeated where the milk/treponeme emulsion was passaged into OTEB+ 10% (*v*/*v*) FCS supplemented with 35 µL rifampicin (5 mg/mL stock), 3.5 µL enrofloxacin (10 mg/mL stock in 1M KOH) and 3.5 µL 1M hydrochloric acid (to balance the pH of the enrofloxacin) to assess the effects on treponeme growth of an antibiotic mixture to suppress growth of other contaminating bacteria.

### 2.9. Treponeme Detection from Fresh Foremilk from DD Symptomatic and Asymptomatic Cows

To investigate the in vivo presence of treponemes in milk, a foremilk was taken immediately prior to milking the cow (*n* = 50) by the dairy farmer and a simplified DD status (acute, chronic, no DD) [30] of the cow recorded by use of an inspection mirror in the parlour. The university dairy farm was used for this sample collection due to the close proximity to the laboratory. Samples were immediately processed and all DNA extractions initiated and all samples inoculated into OTEB 10% (*v*/*v*) FCS within 2 h of milking.

One ml of foremilk from DD symptomatic (*n* = 19) and asymptomatic (*n* = 31) cows was subjected to DNA extraction using the Milk Bacterial DNA Isolation kit (Norgen Biotech Corp., Thorold, ON, Canada) by manufacturer instructions. Extracted DNA was then screened for DD-associated treponemes using the PCR assays described above.

### 2.10. Detection of Treponemes in Fresh Foremilk from DD Symptomatic and Asymptomatic Cows

Here, 0.5 mL of foremilk was added to 7 mL OTEB 10% (*v*/*v*) FCS and incubated at 37 °C in an anaerobic cabinet. At the following timepoints a sample (1 drop) was removed from the liquid culture using a glass pipette and examined under the phase contrast microscope to identify treponeme morphology and assess growth: 24 h, 1 week, 2 weeks, 3 weeks, 4 weeks, 5 weeks and 6 weeks. After 6 weeks, a Chelex extraction was performed on the liquid culture and the extracted DNA screened and analysed with the DD treponeme PCR assays. Cultures were allowed to grow for 6 weeks, as treponemes are fastidious and it can take several weeks for growth to be detected, and it was the time used to assess for DD-associated treponeme survival in a previous study [25].

### 2.11. Statistical Analysis

The data was added to a 2 × 2 contingency table. An online calculator for the Chi squared test was used to assess the difference between groups (https://www.graphpad.com). Significance was set at *p* = 0.05.

## 3. Results

### 3.1. Validation of the DD-Associated Treponeme PCR Assays

The original 12 samples from the pilot study [1], were sourced and retested using the methods in this study (including DNA extraction). The results were the same as the original study with 11/12 positive for a DD-associated treponeme (Supplementary Table S1). The only variation was that one sample (sample 11) in the *T. pedis* phylogroup PCR was negative but was positive in the pilot study (possible due to freeze thaw cycle).

### 3.2. Screening ITN Samples for DD-Associated Treponemes

There were 62 swabs and 33 tissue samples from ITN lesions analysed by the nested DD-associated treponeme PCR assays. In addition, 18 swabs from healthy teats from the ITN positive cows and, when possible, swabs from the teats of a healthy cow from the same farm of a similar age and production stage (*n* = 10) were also screened for DD-associated treponemes. All screening samples from healthy live animals were swabs. Of the 95 ITN lesions, 34 (35.8%) were positive for at least one DD-associated treponemes and only 1 of 18 (5.6%) teats from a non-affected teat from a cow with an ITN lesion were positive (Table 3). No swabs from the age- and production stage-matched asymptomatic animals were positive for DD-associated treponemes using the PCR assays.

When using the Chi squared test there was a statistical difference between the number of ITN teats (tissue and swab samples) positive (34/95) by the *Treponema* genus PCR assay and the number of the non-affected teats positive on the same animal (1/18) (*X^2^*, (1, *N* = 113) = 6.47, *p* = 0.01) (Table 3). There were 95 samples from ITN lesions. Additionally, 18 swabs were obtained from symptomatic cows of teats without ITN lesions; only one (5.6%) was positive for a single DD-associated treponeme phylogroup. There were 20 (21.1%, *n* = 95) ITN samples positive for one DD-associated treponeme phylogroup; four (4.2%) were positive for two DD-associated treponeme phylogroups and 10 (10.5%) were positive for the three recognised DD-associated treponeme phylogroups. There were 34 (35.8%) ITN samples positive for at least one DD-associated treponeme phylogroup.

### 3.3. Detection of DD-Associated Treponemes in Milk

Optimisation of the protocol found that DD-associated treponemes spiked in whole and UHT milk, obtained from the supermarket, could be detected routinely within 2 h of incubation at different temperatures. Additionally, there was no difference in the treponeme growth if the milk/treponeme emulsion was passaged into OTEB 10% (*v*/*v*) FCS with or without antibiotics. The protocol used to investigate DD-associated treponeme presence in foremilk obtained from cows with and without DD lesions excluded antibiotics and samples were processed within 2 h of milking (Table 4).

Looking into the effect of temperature at 37 °C and 4 °C on treponemes’ integrity when incubated in milk for 24 h, no treponeme morphology was observed via phase contrast for either of the groups. After sub-culture, DD-associated treponemes could not be observed by phase contrast microscopy at any time points from 24 h to 6 weeks after inoculation into OTEB 10% (*v*/*v*) FCS and were not detectable using the DD-associated treponeme PCR assays after 24 h of culture. All positive controls retained treponeme morphology and movement across the 6 weeks when examined under phase contrast microscopy. All positive controls samples were also positive for both the *Treponema* genus and the DD *T. phagedenis* phylogroup PCR assays.

### 3.4. Detection of DD-Associated Treponemes in Foremilk from DD Symptomatic and Asymptomatic Cows

Foremilk was collected from 50 cows prior to milking. Nineteen cows had lesions on the hind feet consistent with chronic DD, 31 cows had healthy hind feet (Table 5). More cows with healthy feet without DD had treponemes detected in their foremilk (19.4%) than cows with DD (5.3%) using the *Treponema* genus PCR assay. However, this difference was not statistically significant using the Chi squared test (*X^2^* (1, *N* = 50) = 1.94, *p* = 0.16) and the PCR assays for DD treponemes were negative for all samples.

After inoculation of milk into OTEB 10% (*v*/*v*) FCS, all samples examined using phase contrast microscopy did not detect spirochaetal morphology after 24 h incubation or at any time point in the weekly checks, including at 6 weeks post inoculation. At 6 weeks a DNA Chelex extraction was performed and all samples were negative when assayed using the treponemes PCR assays. All positive controls retained treponeme morphology and movement across the 6 weeks when examined under phase contrast microscopy. All positive controls samples were also positive for both the *Treponema* genus and the DD *T. phagedenis* phylogroup PCR assays at the 6 weeks time point.

## 4. Discussion

### 4.1. PCR Screening of Teat Samples for DD-Associated Treponemes

Clegg et al. [1] suggested that ITN may be another skin disease that is associated with the presence of specific phylogroups of *Treponema* bacteria, due to the detection of DD-associated treponemes by PCR in 11 of 12 (91.7%) ITN lesions from dairy cows in a pilot study. An infectious agent was sought after there was an increase in reported cases from 2004 onwards [3,31]. The original samples from that pilot study were sourced and used to validate the methods in this study. From the screening of the samples collected in the current study, 34/95 (35.8%) teats with ITN lesions were positive for at least one DD-associated treponeme, using similar nested PCR assays. One (*n* = 18, 5.6%) teat without an ITN lesion from a cow with an ITN on another teat was positive for a DD-associated treponeme by PCR. As treponemal bacteria are highly motile, this positive may indicate spread over the skin from the ITN lesion. In the ITN lesions there was predominantly a single DD-associated treponeme phylogroup, compared to 12.4% of samples with two or more different DD-associated treponeme phylogroups. This is in stark contrast to Clegg et al. [1] where 10 (83.3%, *n* = 12) ITN lesions contained two or more DD-associated treponeme phylogroups. Interestingly, in that study, all farms also had cases of DD in the milking herd [1]. The current findings, however, are consistent with more recent published epidemiological findings [3] and the clinical history obtained during sampling, that farms with ITN do not report having current issues with DD lameness in their dairy herd. Two animals with DD lesions and concurrent ITN lesions had both anatomical locations screened for DD-associated treponemes via PCR assays (data not presented). In both of these animals the DD lesions on the hind feet were positive for DD-associated treponemes but the ITN lesions were negative. While the number of animals with concurrent lesions are low, these findings, along with the screening of larger numbers of ITN lesions and the epidemiological data, are beginning to suggest that the DD-associated treponemes may not be a primary pathogen for ITN as once thought. It is possible that the detection of DD-associated treponemes may indicate a higher burden of DD-associated treponemes in the environment, which are contaminating damaged tissue of the teat as a secondary agent [23] or that DD-associated treponemes are present in the lesions at different time points due to differences in the microenvironment of the tissue. Similarly, the non-healing horn lesions of white line disease and sole ulcer can become infected with DD-associated treponemes, making them difficult to treat, but these bacteria are not considered to be the primary aetiological agent [12,32]. Indeed, when it comes to teat skin, the mechanical effects of machine milking on the aetiopathogeneisis requires consideration which may create skin abrasions allowing for infection by bacteria. Potentially, the observed high association of ITN with these taxa in the pilot may result from complicated ITN lesions that do contain DD-associated treponemes, whereas in this study the reduction in association may result from the inclusion of more non-complicated ITN samples.

A similar disease of the udder with relatively low numbers (10%) of samples detected as containing DD-associated treponemes phylogroups via PCR is UCD [20]. The authors of that study indicated that it was likely that UCD was a polymicrobial disease rather than involvement of a single aetiological agent. Given there are similarities between ITN and UCD, in that both affect the skin of the udder, it seems appropriate to investigate the potential polymicrobial nature of ITN. In addition, more recent studies investigating the microbiome of cows with and without DD lesions also suggests that DD may be more of a polymicrobial disease [9,10,11] indicating diseases initially considered likely to be due to a single aetiological agent with recent multi-omics studies now appear as more likely a polymicrobial pathogenesis. It is possible that ITN is a multifactorial and polymicrobial disease involving opportunistic bacteria.

### 4.2. The Assessment of DD-Associated Treponemes in Milk

The dairy environment has been shown to have multiple sites where DD-associated treponemes can be detected. These include cow faeces and slurry, cow footprints, hoof trimming equipment, and especially hoof knives and gloves used during the foot trimming process [25,26,33]. In addition, different types of dairy farm bedding material has been shown to enable survival of DD-associated treponemes in laboratory settings [26]. However, there are only a few investigations into milk as a potential reservoir for DD-associated treponemes. Given that detection of DD-associated treponeme phylogroups via PCR assays are present in a proportion of ITN and UCD cases, it was timely to see if the foremilk and milk could act as a potential reservoir. In fact, DD-associated treponemes have been detected in foremilk from cows with UCD lesions [22]. As the teat canal seals after milking, the teat canal could potentially lead to an anaerobic site suitable for fastidious bacteria such as treponemes to proliferate. However, for treponemes to be able to proliferate in milk they must be able to survive in the substance. Cow’s milk has been shown to have several antimicrobial properties, derived from different peptides within the milk that are effective against both gram-positive and gram-negative bacteria [34]. The findings presented demonstrate that the treponemes spiked in milk and incubated at different temperatures (one to represent body temperature at 37 °C and another to represent refrigeration at 4 °C) could not survive as determined by sub-cultures for 6 weeks. This provided ample opportunity to detect growth if present, as treponeme bacteria are slow growing. Another finding when investigating treponeme growth in milk was there was no difference in treponeme growth when adding a previously documented antimicrobial cocktail to the liquid culture broth at subculture [1], and there was no difference in the high numbers of contaminating bacteria in both cultures (with and without antimicrobials). Consequently, the survival experiments were devised without the antibiotic cocktail for the assessment of treponemes in foremilk. Despite not being able to detect live spirochaetal bacteria via phase contrast microscopy at weekly intervals during the milk survival experiments, the treponeme bacteria can be readily detected via PCR assays up to 2 h after inoculating the bacteria into milk. Whilst this demonstrates the presence of non-degraded treponemal DNA rather than live bacteria it, while speculative, theoretically could allow for infection to transmit to a calf or another human. Also, there is the hypothesised potential to transmit to the next cow to be milked in the parlour if the teat cups are not cleaned between cows during milking. However, further studies are required to determine this. In reality, milk will be stored in the bulk milk tank and transported and processed more for than 2 h before there is any potential for the public to ingest milk, and the treponeme bacteria are likely to have degraded enough to not cause a risk for human ingestion. In addition, these data also suggest the possibility of DD-associated treponeme phylogroups being detected in foremilk from cows with DD is unlikely. Therefore, milk and foremilk do not seem to be a viable infection reservoir for DD-associated treponeme bacteria. However, these data contrast the results of Sobhy et al. [22] and therefore we suggest further studies are required to clarify any associations with local husbandry and bedding environment on milk and UDC presence, as these might explain the differing results across these various geographical regions. This is especially important given the contrasting substantial impact of different beddings on DD-associated treponeme survival [26]. When looking at foremilk samples from cows with and without DD lesions it was demonstrated that, while none had the pathogenic DD-associated treponemes detected, there were a higher proportion of cows with healthy feet positive for treponeme genus via PCR than the cows with DD lesions. Whilst this result was not significant, it contributes to the theory that there are non-pathogenic treponemes present in the dairy cow microbiome. Such a non-pathogenic treponeme *T. ruminis* sp. nov has been found in the gut microflora [35] and it is possible that there are as yet unidentified non-pathogenic treponemes present in the skin microflora, which is another area for future study.

## 5. Conclusions

From the data presented it seems unlikely that DD-associated treponemes are the primary infectious agent involved in the development of ITN lesions. Given only a subset of ITN lesions had DD-associated treponemes present, ITN is likely a multifactorial and polymicrobial disease. Further investigations are required in these areas to aid in the understanding of the development of these lesions.

DD-associated treponemes do not seem to be able to be detected for longer than 2 h in milk and foremilk samples when incubated at 37 °C or 4 °C. Moreover, all milk samples we surveyed were DD-associated treponeme negative, therefore we do not consider that DD-associated treponemes reside within the udder milk, or are transmitted by milk expulsion from the teat canal.

## Figures and Tables

**Table 1:** PCR assay primers for DD-associated treponeme screening.

Primer	Primer Sequence (5′-3′) (Forward and Reverse)	16S rRNA Gene Position	Band Size (bp)	Reference
Universal 16S rRNA gene	AGAGTTTGATCCTGG TACCTTGTTACGACTT	7–26 1491–1506	1526	[28]
*T. medium* phylogroup	GAATGCTCATCTGATGACGGTAATCGACG CCGGCCTTATCTAAGACCTTCTACTAG	472–500 1001–1029	475	[6]
*T. phagedenis* phylogroup	GAAATACTCAAGCTTAACTTGAGAACTTGC CTACGCTACCATATCTCTATAATATTGC	612–640 1006–1029	400	[6]
*T. pedis* phylogroup	GGAGATGAGGGAATGCGTCTTCGATG CAAGAGTCGTATTGCTACGCTGATATATC	459–484 1017–1045	475	[6]
*Treponema* genus	AARCATGCAAGTCGARCGGCAAG TCCATTGCGGAATATTCTTA	49–71 365–384	335	[29]

**Table 2:** PCR cycling conditions for universal bacterial 16S rRNA gene, *Treponema medium* phylogroup, *Treponema phagedenis* phylogroup, *Treponema pedis* phylogroup and *Treponema* genus specific 16S rRNA gene.

	Initial Denaturation	Denaturation	Annealing	Elongation	Final Elongation
Universal bacterial 16 S rRNA gene [28]					
Temperature	95 °C	94 °C	55 °C	72 °C	72 °C
Time	5 min	1 min	3 min	3 min	7 min
Cycles	1	24			
*Treponema medium* phylogroup [6]					
Temperature	95 °C	95 °C	68 °C	72 °C	72 °C
Time	5 min	1 min	1 min	2 min	10 min
Cycles	1	39			
*Treponema phagedenis* phylogroup [6]					
Temperature	95 °C	95 °C	64 °C	72 °C	72 °C
Time	5 min	1 min	1 min	2 min	10 min
Cycles	1	39			
*Treponema pedis* phylogroup [6]					
Temperature	95 °C	95 °C	68 °C	72°C	72 °C
Time	5 min	1 min	30 s	2 min	10 min
Cycles	1	39			
*Treponema genus* specific 16S rRNA gene [29]					
Temperature		95 °C	64 °C	72 °C	72 °C
Time		30 s	1 min	1 min	10 min
Cycles		40			

**Table 3:** Summary of PCR screening of ITN samples for DD-associated treponeme bacteria phylogroups.

Sample (ITN +/− Teat and +/− Cow)	Sample Type (Swab/Tissue)	Number	*Treponema* Genus	Group 1	Group 2	Group 3
ITN positive teat	Swab	62	21/62 (33.8%)	12/62 (19.4%)	10/62 (16.1%)	17/62 (27.4%)
ITN positive teat	Tissue	33	13/33 (39.4%)	4/33 (12.1%)	6/33 (18.2%)	10/33 (30.3%)
Total ITN positive teat samples (both swabs and tissue)	Swab and Tissue	95	34 (35.8%)	16 (16.8%)	16 (16.8%)	27 (28.4%)
Asymptomatic teat from ITN positive cow	Swab	18	1/18 (5.6%)	0	0	1/18 (5.6%)
Matched ITN negative cow	Swab	10	0	0	0	0

+ indicates a positive case or animal, − indicates a negative case or animal, ITN—ischaemic teat necrosis, DD—digital dermatitis, Group 1—DD *Treponema medium* phylogroup, Group 2—DD *Treponema phagedenis* phylogroup, Group 3—DD *Treponema pedis* phylogroup.

**Table 4:** Summary of the length of time treponemes can be detected via the nested PCR assays for DD-associated *Treponema phagedenis* phylogroup and *Treponema* genus.

	Time							
PCR Assay	10 min	30 min	1 h	2 h	4 h	8 h	24 h	Negative Control
*T. phagedenis*	+	+	+	+	−	−	−	−
*Treponema* genus	+	+	+	+	+	+	−	−

+ indicates a positive result on PCR and − indicates a negative result on PCR.

**Table 5:** DD treponeme PCR results from foremilk samples.

Foremilk Sample	Number	*Treponema* Genus	Group 1	Group 2	Group 3
Cows with healthy hind feet	31	6 (19.4%)	0	0	0
Cows with DD lesions on hind feet	19	1 (5.3%)	0	0	0

Group 1—DD *Treponema medium* phylogroup, Group 2—DD *Treponema phagedenis* phylogroup, Group 3—DD *Treponema pedis* phylogroup.

## Data Availability

No new data were created or analyzed in this study. Data sharing is not applicable to this article.

## Supplementary Materials

The following supporting information can be downloaded at: https://www.mdpi.com/article/10.3390/pathogens13050427/s1, Table S1: Validation results for the DD treponeme PCR assays using the samples obtained in the pilot study.
