# Supplementary material for: Molecular Screening for Digital Dermatitis-Associated Treponemes in Bovine Ischaemic Teat Necrosis Lesions and Milk in Dairy Cattle
Source: Pathogens. 2024 May 17;13(5):427. doi: 10.3390/pathogens13050427 (PMC11123857; doi:10.3390/pathogens13050427)
Supplement: Supplementary file 1 [file pathogens-13-00427-s001.zip › pathogens-2945905-supplementary.pdf]

Supplementary Material

Supplementary Table S1. Validation results for the DD treponeme PCR assays using the samples obtained in the pilot study.

Pilot study samples	*Treponema* genus	Group 1	Group 2	Group 3
1	+	+	+	+
2	+	-	+	+
3	+	-	+	+
4	+	-	+	-
5	+	+	+	+
6	+	+	+	-
7	+	-	+	+
8	+	+	+	-
9	+	+	+	+
10	+	-	+	-*
11	+	+	+	-
12	-	-	-	-

*+ indicates a positive case or animal, - indicates a negative case or animal, Group 1- DD Treponema medium phylogroup, Group 2 – DD Treponema phagedenis phylogroup, Group 3 – DD Treponema pedis phylogroup. * - variation from the pilot study.*
